# Supplementary material for: Tumor Progression Reverses Cardiac Hypertrophy and Fibrosis in a Tetracycline-Regulated ATF3 Transgenic Mouse Model
Source: Cells. 2023 Sep 15;12(18):2289. doi: 10.3390/cells12182289 (PMC10528851; doi:10.3390/cells12182289)
Supplement: Supplementary file 1 [file cells-12-02289-s001.zip › cells-2588877-supplementary.pdf]

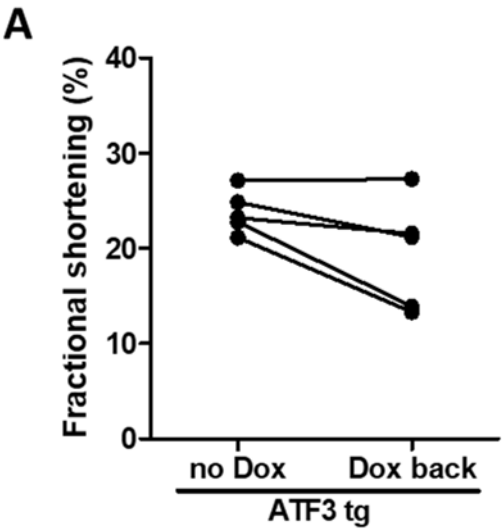

Figure S1: Cardiac contractility function is mildly affected after Dox addition**.**

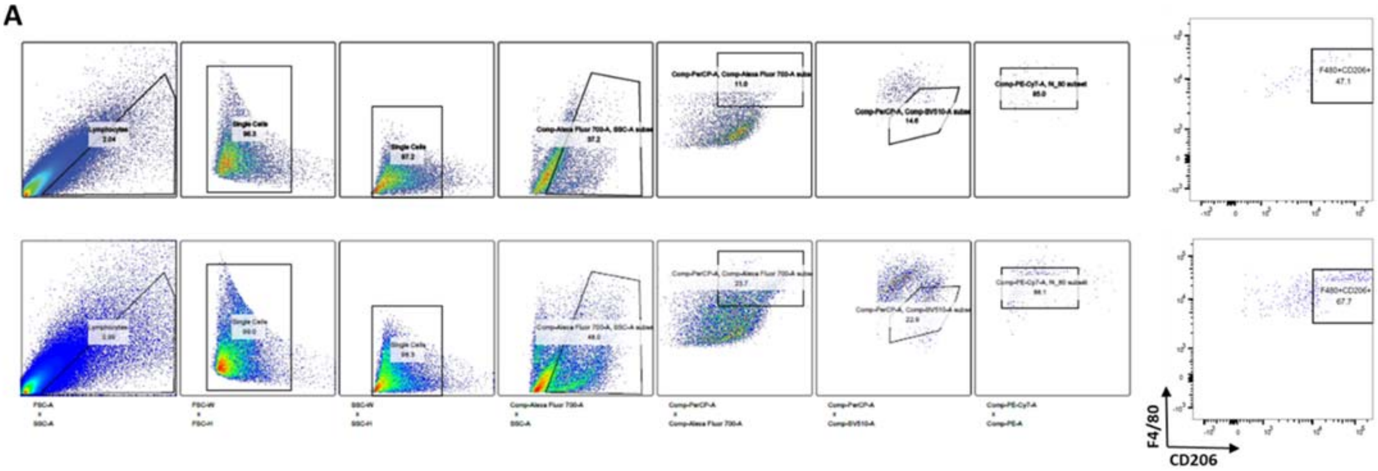

Figure S2: Flow cytometry gating strategy for identification of cardiac macrophages.

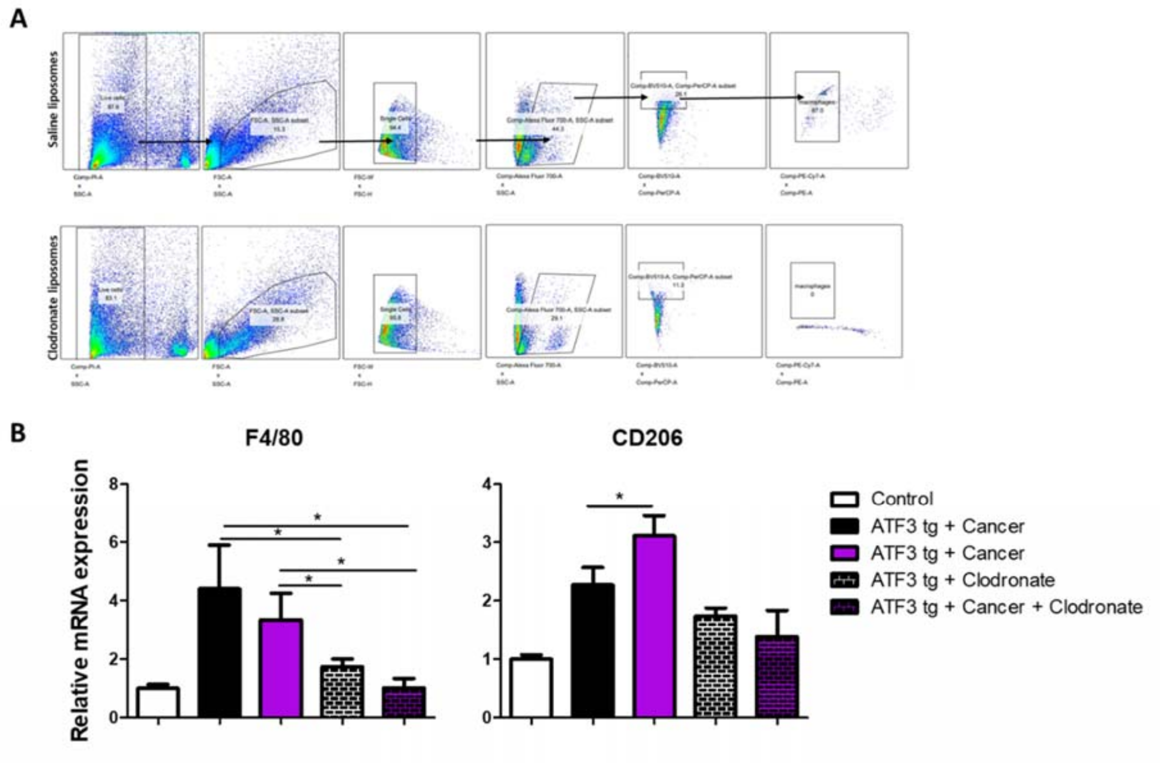

Figure S3: Clodronate containing liposomes lead to significant depletion of cardiac macrophages.

Table S1: Oligonucleotides sequences.

**Gene**	**Forward**	**Reversed**
Hsp90	TCGTCAGAGCTGATGATGAAGT	GCGTTTAACCCATCCAACTGAAT
ANP	GCTTCCAGGCCATATTGGAG	GGGGGCATGACCTCATCTT
BNP	GAGGTCACTCCTATCCTCTGG	GCCATTTCCTCCGACTTTTCTC
TGFβ3	CCTGGCCCTGCTGAACTTG	GACGTGGGTCATCACCGAT
CTGF	AGACCTGTGGGATGGGCAT	GCTTGGCGATTTTAGGTGTCC
F4/80	CCCCAGTGTCCTTACAGAGTG	GTGCCCAGAGTGGATGTCT
CD206	CTAACTGGGGTGCTGACGAG	GGCAGTTGAGGAGGTTCAGT
CD163	CCTCCTCATTGTCTTCCTCCTGT[illegible]	CATCCGCCTTTGAATCCATCTCTT[illegible]
TNFα	CCCTCACACTCAGATCATCTTCT	GCTACGACGTGGGCTACAG
CCL2	GTGATGGAGGGGGTCAGGA	GGGATGGGACAGCCTAAACT

Table S2: Average of Echocardiography parameters of Figure 1B.

	LVID;d	LVID;s	FS%
ATF3 tg	3.770125	2.871625	23.79346
ATF3 tg+Cancer	3.983857	2.83	29.06459

Table S3: Average of Echocardiography parameters of Figure 4B.

	LVID;d	LVID;s	FS%
ATF3 tg	3.5975	2.855	20.5225
ATF3 tg+Cancer	3.674	2.602	29.154
ATF3 tg + Clodronate	3.7775	2.9875	20.9225
ATF3 tg + Cancer + Clodronate	3.792	3.012	20.494
